# Reasons for migration, parental acculturation, and language: the case of Chinese American and Mexican American parents and dual language learners

**DOI:** 10.3389/fpsyg.2023.1237143

**Published:** 2023-09-04

**Authors:** Maria Belen Buttiler, Qing Zhou, Yuuko Uchikoshi

**Affiliations:** ^1^School of Education, University of California, Davis, Davis, CA, United States; ^2^Department of Psychology, University of California, Berkeley, Berkeley, CA, United States

**Keywords:** immigration, migration reasons, acculturation, cultural orientation, language input, dual language learners, vocabulary

## Abstract

Migration is a complex process associated with a range of social, economic, and political reasons. In the U.S., almost one-quarter of the total population of parents are immigrant parents of children ages 0–10. Immigrant parents transmit values from their culture of origin as well as their language to their children. Additionally, they may undergo a process of cultural and psychological change known as acculturation. Research has shown that acculturation can be linked to parenting styles and adolescents’ psychological well-being and behavioral problems. However, little is known about the associations among immigrant parents’ acculturation, their home language and literacy practices, and their bilingual children’s language skills. This study explores the relationships among reasons for migration, parental acculturation, home language and literacy practices, and child expressive vocabulary in English and their heritage language (HL). A group of 190 Spanish-English (*N* = 66) and Chinese-English (*N* = 124) dual language learners (DLLs) (mean age = 48.98 months) and their Chinese and Mexican parents (mean age of migration = 18.57 and 21.38 years old respectively participated. Frequency counts revealed that Mexican American families migrated to the U.S. mostly for multiple reasons, including joining family members, getting married, and looking for better education or job opportunities, whereas most Chinese American families migrated for family reasons only. Path analysis models showed that, for both cultural groups, language input in Spanish and Chinese mediated the relationship between parents’ cultural orientations and DLLs’ HL expressive vocabulary. These findings emphasize that despite the heterogeneity of immigrant families and the variability in DLLs’ vocabulary skills in preschool, there exist some similarities across immigrant parents and bilingual children. A deeper understanding of acculturation practices and home language use can help educators better support children from diverse backgrounds and promote cultural awareness and sensitivity in the classroom.

## Introduction

During the first 5 years of life, children show rapid growth in their physical, cognitive, and social–emotional domains, and the interactions and experiences that they have during this period lay the foundation for their future development and well-being (Center on the Developing Child, 2007). Parents are usually children’s first social partners and, as such, play an essential role in their children’s first years of development (Mendelsohn et al., 2011). Immigrant parents, in particular, transmit knowledge and values from their culture of origin and language to their children (e.g., Bornstein, 2009; Baker, 2016). In the U.S., almost one-quarter of the total population of parents are immigrant parents of children ages 0–10 (Hofstetter and McHugh, 2021). Reasons for migration may vary depending on social or family connections, economic or employment opportunities, educational goals, and political motives (e.g., Giuntella et al., 2018; Czaika and Reinprecht, 2022). Individual motivations for migration can be complex and multifaceted. Since migrants may face significant challenges as they transition to a new country, learn a new language, and navigate a new school system, their children can be affected by cultural adaptation stress (Good et al., 2010). Stressors such as economic hardship, poor health, and lack of emotional or emergency support are commonly experienced by foreign-born Hispanic couples in the U.S. (Wildsmith and Chen, 2023).

As immigrants make contact with and engage in new cultures, they undergo cultural and psychological change known as acculturation (Berry, 2005, 2019). Berry’s acculturation model (Berry, 1992) classifies change into two dimensions. The first one considers whether and to what degree individuals choose to preserve or abandon their culture of origin, reflecting the importance they attach to maintaining their identity and unique traits. The second dimension focuses on whether individuals choose to embrace or distance themselves from the host (or receiving) culture, indicating the significance they place on establishing connections with the broader society. Acculturation may include conscious and unconscious choices regarding social relationships, language use, media preferences, and attachments to cultural groups. Language use and language choices may vary among bilingual and multilingual families, ranging from a dominance of the caregivers’ heritage language (HL) to a dominance of the language of the receiving culture (e.g., English in the U.S.). Societal and political factors such as immigration policies, attitudes toward immigrants, immigration status, and economic inequality can influence these choices (Schwartz et al., 2010; Suárez-Orozco et al., 2018). Furthermore, the acculturation process may vary among people, as there are individual and group differences in how immigrants adopt and employ acculturation strategies (Berry, 2019). Among family members, acculturation often takes place at different rates and with different aims (Berry, 2005). Acculturation may be influenced by the length of stay in the receiving country as well as individuals’ age at the time of migration, socioeconomic status, and the desire to assimilate into the dominant culture (Hsiung and Ferrans, 2007). Hence, it is important to examine how the acculturation of immigrant caregivers from different cultural groups may be related to their language use and language choices, and their children’s language outcomes.

In the U.S., an increasing number of immigrant families and young children are exposed to more than one language and culture in their homes, communities, and schools (Park et al., 2022). This calls for educators, school administrators, and policymakers to better understand this diverse scenario. Previous research has primarily examined the acculturation of immigrant parents and their adolescent children, focusing on factors including psychological well-being, depressive symptoms, behavioral problems, and parenting styles (e.g., Liu et al., 2009; Costigan and Koryzma, 2011; Kim et al., 2015). Only a few studies have examined the relationship between parental acculturation and the oral proficiency of dual language learners’ (DLLs’)–children 0–8 who are learning two or more languages (e.g., Tsai et al., 2012; Troesch et al., 2021; Uchikoshi et al., 2022). Little is known about the relationships among the reasons for migration, acculturation, and the home language practices of immigrant parents of young children living in the U.S. Given the cultural, social, and linguistic heterogeneity of immigrant families, the development of language and literacy among DLLs can vary widely. Yet, another underexplored area is how these family characteristics and practices are associated with DLLs’ expressive vocabulary in English and their HL. Hence, the purpose of this study was to identify the main reasons for migration of a group of 190 Chinese American and Mexican American parents living in the U.S. and to explore the role of parental acculturation and home language and literacy practices in both languages in the pathways to predict preschooler DLLs’ expressive vocabulary in English and their HL (i.e., Chinese or Spanish).

### Theoretical frameworks

Given the importance of understanding the social contexts and environmental factors that play a role in DLLs’ language development, this study is informed by the integrative risk and resilience model for understanding the adaptation of immigrant-origin children and youth (Suárez-Orozco et al., 2018). This framework emphasizes developmental tasks (e.g., building self-regulation skills and social relationships), psychological adjustments (e.g., well-being and distress), and acculturative tasks (e.g., developing cultural competence and ethnic identities) that are specific to immigrant and immigrant-origin children and youth. In addition to these individual-level factors, this model considers families as a microsystem within which parental practices and cultural beliefs, support, and family separations and reunifications play a crucial role in the developmental domains of children. Moreover, this conceptual model considers global economic, geopolitical, and social dynamics that have direct implications for migrant families, such as economic inequality, immigration policies, and attitudes toward immigrants. Consequently, children’s developmental skills are shaped by social, political, and economic factors that are unique to immigrant and immigrant-origin families.

To explain the relationship between home literacy practices and children’s language outcomes, this study is grounded in Sénéchal et al. (1998) home literacy model, which emphasizes the role of home literacy practices and activities in shaping young children’s oral language and early literacy skills. According to this model, storybook exposure and parent teaching about literacy will likely improve children’s oral language skills, which may influence children’s literacy skills. A large body of literature supports the home literacy model. Previous studies have demonstrated the relationships between home literacy and children’s vocabulary growth and oral language development (e.g., Hood et al., 2008; Sénéchal and LeFevre, 2014; Mak et al., 2023). Yet, previous research studies have also shown that the relationships between home literacy practices and children’s vocabulary outcomes vary depending on the children’s developmental stage (e.g., Torppa et al., 2007; Sénéchal and LeFevre, 2014), as well as their sociocultural and linguistic backgrounds (Kalia and Reese, 2009; Hammer et al., 2014).

De Houwer’s (1999, 2007) three-tier model of bilingual development complements the previous models and explains the role of parental attitudes and beliefs in their language use, which is linked to their children’s language skills. Children’s use of one or two languages in their interactions with their parents is tied to different aspects of their parents’ linguistic behavior. The wide variation in parents’ linguistic behavior results from factors including parental beliefs about how children acquire languages, the parents’ role in that acquisition process, and parents’ attitudes toward a particular language, bilingualism, and patterns of language choice. Taken together, these frameworks provide elements to better understand how specific experiences and aspects of the child’s individual and family environments influence development. Given that the characteristics of the context, the interactions, and the cultural and psychological adjustment of DLLs may differ from those of the English monolingual child population, these conceptual models provide one lens for contextualizing factors that are associated with bilingual children’s language skills in early childhood.

### Families’ reasons for migration to the United States

International migration is defined as the relocation of individuals to another country, leading to temporary or permanent resettlement (Bartram et al., 2014). There exist multiple reasons why individuals migrate to other countries. Migration is a universal characteristic of human history and a social phenomenon associated with a range of life domains, including politics, economic inequality, and variations in political freedom or underrepresentation (Martin et al., 2006). For instance, spatial and structural disparities leading to long-term socio-economic inequalities and gaps in living standards may be the drivers that enable or constrain people’s decisions or destinations. As Bartram and colleagues explained, an immigrant in any given country is often noticeable for being “foreign” and “different” from those born in that country, even if they migrated within the country. Eventually, these cultural exclusions become part of the driving force for new socio-political inequities. In a recent review of the literature on migration drivers, Czaika and Reinprecht (2022) concluded that worldwide research on economic and sociocultural motivations outnumber the other driver dimensions.

The U.S. has the largest number of foreign-born individuals compared to any other country in the world. Over 25 percent of all global migrants (50.6 million people) live in this country (U.S. Census Bureau, 2020). After examining U.S. immigration data from the 1990s, Lobo and Salvo (1998) reported that between 1992 and 1994 immigration of Asian professionals to the U.S. was related to immediate relatives (29.7%), family preferences (26.2%), employment (15.5%), and political refuge (14.7%), among other reasons (13.8%). Later studies have shown that educational and employment opportunities are the primary reasons for migration among Chinese immigrants (Chen et al., 2009). However, there is a dearth of empirical studies investigating the reasons for migration among parents of young DLLs. A more recent study looked at Chinese immigrant parents of 1st- and 2nd-grade children living in the U.S. (Wang et al., 2023). The results showed that parents’ main reasons for migration were to join family members in the U.S., to provide their children with better opportunities–including education–, and because a family member brought them to the country. Additionally, the authors observed that families who migrated solely to seek better educational and job opportunities reported having significantly higher education and income levels than parents who migrated for family reasons or a combination of family and other reasons.

Although Mexicans remain the largest immigrant population in the U.S. (comprising around 24 percent of the 45.3 million foreign-born residents as of 2021), their numbers have decreased in the last ten years. Since 2013, India and China have been the top countries of origin for new immigrants to the U.S. However, Rosenbloom and Batalov (2022) reported that, during the pandemic, Mexicans have again become the largest new immigrant group. The researchers also showed that the immigration pathways of Mexican Legal Permanent Residents in the U.S. were related to (1) having immediate relatives of U.S. citizens (70%), (2) having employment opportunities (6%), and (3) seeking refuge and asylum (1%). Previous studies have explored the relationship between migrant parents living in the U.S. and children’s educational aspirations while still in Mexico, revealing that having a migrant mother can positively predict children’s educational aspirations compared to children with no migrant parents (Dreby and Stutz, 2011). Yet, more research is needed to understand how reasons for migration may relate to parents’ acculturation and their home language and literacy practices.

Similarly, more research is needed to explore family characteristics and practices that can influence the language development of DLLs from culturally and linguistically diverse immigrant backgrounds. Hence, the following sections summarize existing literature in the field of bilingualism and cultural studies that explains the relationship between DLLs’ language outcomes and parental acculturation, home literacy practices, language use (e.g., Tsai et al., 2012; Bitetti and Hammer, 2016; Uchikoshi et al., 2022; Mak et al., 2023).

### Parental acculturation and child language skills

Families play a crucial role within the microsystems in which children develop. In the case of DLLs in particular, these microsystems contain multicultural contexts of socialization where children and their families navigate everyday routines between the home culture and the host culture (Suárez-Orozco et al., 2018). Immigrant and immigrant-origin parents teach children about their ethnic background and promote national costumes and racial pride. Some examples may include talking about important historical figures and events, sharing culturally relevant books, music, and stories, celebrating holidays from the culture of origin, and encouraging children’s use of their heritage language (Hughes et al., 2006). The differential geographical distribution can also play a role in preserving the culture of origin. Bornstein and Cote (2006) cite the example of Latinos living in states with higher numbers of Latino individuals, such as California, Texas, New York, and Florida, for whom it may be easier to retain costumes from their culture of origin and socialize with people from their home country or people who speak the same language.

Immigrant families may struggle to balance maintaining their cultural heritage and adapting to a new culture while raising their children. This can include changes in parent–child interactions (e.g., parenting practices and language use), which are likely to influence children’s health and development (Bornstein and Cote, 2006). Therefore, a better understanding of these changes and how they relate to DLLs’ home environment and language development is relevant to early education programs. Preschool teachers working in multilingual classrooms already use effective language strategies to help students thrive (Chan et al., 2022). Thus, knowledge about immigrant families’ home cultures and literacy activities can also inform teachers’ pedagogical approaches. Yet relatively little is known about the relationships among parental acculturation, language input, home literacy practices, and DLLs’ oral language development.

Previous studies have explored the associations between immigrant parents’ cultural orientations^1^ and children’s bilingual skills at different ages (Tsai et al., 2012; Cote and Bornstein, 2014; Baker, 2016; Troesch et al., 2021; Uchikoshi et al., 2022), and a few of these studies have shown different findings. Cote and Bornstein (2014) found an indirect link between maternal acculturation and children’s vocabulary size in the heritage and societal language among children from Latino, Japanese, and Korean immigrant groups. Maternal acculturation was also found to positively predict the amount of input that children received in each language. Tsai et al. (2012) found indirect relations between parental ethnic-cultural orientation and their children’s Chinese language skills at ages 4–7. The parents’ use of Chinese mediated the relations. Moreover, parents’ use of Chinese, rather than general cultural maintenance values, was associated with children’s expressive Chinese proficiency and, to a lesser extent, with receptive proficiency. These findings suggest that at-home HL use, instead of cultural maintenance, is linked to children’s HL development.

More recently, Troesch et al. (2021) conducted a study of immigrant families in Switzerland with preschool-age children speaking German as a second language. The authors found that parental acculturation attitudes toward the country of origin negatively correlated with children’s German skills. In a recent study conducted with pilot data, our research team examined the links between Mexican American and Chinese American parental acculturation and bilingual preschoolers’ language abilities (Uchikoshi et al., 2022). In this study, parental acculturation levels were similar among DLLs with four distinct bilingual abilities–high proficiency in both languages, low proficiency in both languages, English-dominant, and Spanish or Cantonese-dominant. The results also showed that parents of English-dominant children had significantly higher levels of American identity than parents of bilingual children with relatively high proficiency in both languages.

### Language input, home literacy practices, and child’s language skills

In the U.S., one in three children ages five and under lives in a household where a language other than English is spoken by a parent or caregiver (Park and Pompa, 2021). The linguistic experiences of DLLs vary widely depending on when children were exposed to each language, the quantity of exposure to each language, and the quality of language input (Hammer et al., 2014; National Academies of Sciences, Engineering, and Medicine, 2017). As DLLs start going to school, they may shift their dominant language from their HL to the host country’s dominant language–for example, English in the U.S. (Luykx, 2005). Some children may also experience HL attrition, losing their bilingual abilities by the time they reach adolescence or young adulthood (Portes and Rumbaut, 2006; Nesteruk, 2010). This can be the result of immigrant parents discouraging their children’s HL use out of fear of discrimination or limited academic and economic opportunities (e.g., Suárez-Orozco et al., 2018). Consequently, DLLs’ cultural identity and family relationships may be negatively affected (Wong Fillmore, 2000; Ennser-Kananen, 2012).

Exploring the language input and home literacy practices of DLLs from diverse cultural backgrounds is important to better understand the unique language and literacy experiences of these children and their caregivers. A variety of home literacy practices including book reading, storytelling, knowledge of the alphabet, and attention to print have been explored in previous studies with monolingual and bilingual children (e.g., Dickinson and Tabors, 2001; Neuman and Dickinson, 2011). Researchers have found strong relations among the frequency of caregiver-child book reading, library visits, and DLLs’ English language outcomes and emergent literacy skills (e.g., Uchikoshi, 2006; González and Uhing, 2008; Hammer et al., 2010; Dixon et al., 2012; Bitetti and Hammer, 2016). The frequency of home literacy activities between mothers and their DLL preschool-age children has been shown to predict children’s English vocabulary and reading abilities in kindergarten in the U.S. (Hammer et al., 2010). In a study conducted with parents and DLLs ages 50–88 months, shared book reading was positively associated with children’s HL oral proficiency (Mak et al., 2023). However, research that specifically investigates the acculturation, home literacy practices, and language use of immigrant families and DLLs is scarce, especially among Spanish-English and Chinese-English young DLLs–the two fastest-growing and largest DLL groups in the U.S. (Budiman and Ruiz, 2021).

### Present study

The current study aims at gaining a better understanding of the main reasons for migration, and examining the complex interplay among parental acculturation, language use, and the home literacy practices of Mexican American and Chinese American families, and how these relate to DLLs’ English and HL expressive vocabulary skills, i.e., Spanish and Chinese. Therefore, the present study investigates the following research questions:

1. What are the main reasons Mexican American and Chinese American parents in this study migrated to the U.S.? Are there any differences between the two cultural groups?
2. Are these reasons for migration associated with Mexican American and Chinese American parents’ acculturation, home literacy practices, and DLLs’ language input?
3. Do parental acculturation, home literacy practices, and DLLs’ language input play a significant role in the pathways to DLLs’ expressive vocabulary in English and Spanish/Chinese during preschool?

To explore these relationships, a path analysis framework was employed, allowing for a comprehensive examination of direct and indirect effects. By utilizing path analysis, this study aims to provide a deeper understanding of the mechanisms through which parental acculturation influences DLLs’ oral language skills, with language input and home literacy practices serving as potential mediators, a relationship that has received limited attention in previous research. By decomposing the total effect of parental acculturation strategies on DLLs’ expressive vocabulary into direct and indirect pathways, path analysis helps to elucidate the specific mechanisms through which the acculturation of two cultural groups of immigrant parents operates. Additionally, path analysis provides a graphical representation of the hypothesized model, making it easier to conceptualize and communicate complex relationships.

Based on prior research, we hypothesized that immigrant parents’ American and heritage cultural orientations would be positively associated with DLLs’ language input (i.e., parents’ language use at home), which would predict children’s expressive vocabulary. Since, to the best of our knowledge, there is no previous research on the link between parents’ cultural orientations and home literacy practices, no hypotheses were made. Yet, based on previous studies and the theoretical frameworks described above, we hypothesized that home literacy practices would predict DLLs’ expressive vocabulary.

## Methods

### Participants

A total of 190 DLLs (66 Mexican American, 124 Chinese American) from low-income immigrant families and their parents participated in this study. The families were recruited from federally- and state-funded preschools in Northern California during parent meetings, school events, and drop-off/pick-up times. The Chinese American families reported speaking four different Chinese dialects at home: Cantonese (76%), Mandarin (13%), Taishanese (9%), and Longdu (2%). The Mexican American children were 36–63 months old (*M* = 48.95; *SD* = 6.61) at the time of data collection, whereas the Chinese American children were 36–61 months old (*M* = 47.99; *SD* = 7.52). Mexican American parents’ age of migration ranged from 2 to 37 years old (*M* = 21.38; *SD* = 8.02), and Chinese American parents’ age of migration ranged from 1 to 40 (*M* = 18.57; *SD* = 13.96). On average, from the year parents migrated to the U.S. to the year in which the data collection took place, Mexican American parents’ length of stay was 15.31 years (range = 3–27, *SD* = 6.70), whereas Chinese American parents’ length of stay in the U.S. was 8.89 years (range = 1–38, *SD* = 6.80). The average level of formal education for Mexican parents was 11.89 years (range = 8–18; *SD* = 3.11), and the average level of formal education for Chinese parents was 13.11 years (range = 8–23; *SD* = 2.86). The descriptive statistics of the participants’ demographic variables are shown in Table 1. There were no statistically significant differences in children’s age between the two groups (*p* > 0.05). Significant differences were found in parents’ age of migration (*t* = −3.90, *p* < 0.001), parents’ length of stay (*t* = 5.43, *p* < 0.05), and parents’ years of formal education (*t* = −2.82, *p* < 0.01). Compared to Mexican parents, Chinese parents were, on average, almost 3 years younger (2.81) when they migrated to the U.S. It is important to notice, that the variability in parents’ age of migration was higher among Chinese parents. Compared to Chinese parents, Mexican parents had lived in the U.S. an average of 6.42 years more than Chinese parents at the time of data collection. Compared to Mexican parents, Chinese parents had, on average, 1.22 more years of formal education.

**Table 1:** Descriptive statistics of the participants’ demographic variables.

	Mexican American (*n* = 66)				Chinese American (*n* = 124)			
	*M*	SD	Min	Max	*M*	SD	Min	Max
Child age (in months)	48.98	6.61	36	63	47.99	7.52	36	61
Parent age of migration	21.38	8.02	2	37	18.57	13.96	1	40
Parent length of stay	15.31	6.70	3	27	8.89	6.80	1	38
Parent years of formal education	11.89	3.11	8	18	13.11	2.86	8	23

### Procedures

First, bilingual research staff contacted site supervisors to coordinate recruitment dates and times. Then, bilingual research assistants visited the preschools to distribute project flyers and collect contact information from the caregivers. Participants were selected based on the following criteria: (a) the child is between 36–71 months old; (b) the child attends a Head Start center or a state-funded preschool at least 3 days per week; (c) the child understands and speaks some English and Cantonese, Mandarin, or Spanish; and (d) both parents identify as ethnically Chinese or Mexican.

The data used in this study are part of a larger, longitudinal project that investigates the language and social–emotional skills of DLLs from low-income immigrant families. The project procedures were approved by the Institutional Review Boards (IRB) at the authors’ research institutions. The data were collected from the fall of 2018 to the spring of 2020. Parents signed informed consent forms before participating. Bilingual research assistants visited the families’ houses, asked the parents to complete a set of questionnaires, and conducted language assessments with the children. The parents received USD 150 compensation for their time, and the children received a book, a puzzle, stickers, and a small prize.

### Measures

#### Reasons for migration

Questions about the families’ reasons for migration were part of the Family Demographics and Migration History Questionnaire (Roosa et al., 2008), which was administered in the parents’ preferred language during the parent interviews. Parents reported their answers based on a list containing 10 reasons for migration (item #10 being “other,” where parents could specify other purposes or motivations). As done in a previous study using the same scale (Wang et al., 2023) and to facilitate analysis and interpretation, participants’ answers were organized into three categories: (a) “family reasons” (e.g., joining family members in the U.S., getting married), (b) “betterment reasons” (e.g., leaving for political and/or personal problems, providing children with better education and opportunities, finding a better job, earning a better income), and (c) “multiple reasons” (a combination of “family” and “betterment” reasons as well as “other” reasons). Parents were asked to mark as many answers as applied to them.

#### Parental acculturation

The parent version of the Cultural and Social Acculturation Scale (CSAS; Chen and Lee, 1996) was used to measure parents’ American and Mexican or Chinese cultural orientations. Since all the acculturation measures were highly correlated, composite scores were created for *American Cultural Orientation* (12 questions) and *Chinese* or *Mexican Culture Orientation* (12 questions) for each cultural group. This parents’ self-reported questionnaire contained questions about parents’ social connections and friends (5-point Likert scale; six questions about the number of friends from each culture and the frequency with which parents invite friends to their house or are invited by friends from either culture), media usage in English and Spanish or Chinese (6-point Likert scale; 10 questions about the frequency with which parents read the newspaper, listen to the radio, watch TV, and listen to music in either language), and language proficiency in English and Spanish or Chinese (4-point Likert scale; eight questions about parents’ self-reported speaking, listening, reading, and writing abilities in each language). The higher the American Cultural Orientation score, the more likely parents are to have adopted aspects of the receiving culture, whereas the higher the Mexican or Chinese Cultural Orientation score, the more likely parents are to have retained values and traditions from their culture of origin. Both scores are not mutually exclusive. This scale has been used in previous studies with Chinese and Mexican immigrants (Chen et al., 2014, 2015; Chen and Zhou, 2019; Uchikoshi et al., 2022).

#### Home literacy practices

Using a 5-point Likert scale, parents reported on the frequency of shared book reading, the frequency of helping their children learn numbers, letters, and words, and the frequency of oral storytelling in both languages, i.e., English, and Spanish or Chinese. The possible answers were: 1 = Almost never, 2 = Once a month, 3 = 2–3 times a month, 4 = 1–2 times a week, 5 = Daily. This questionnaire was adapted from a study conducted by Hammer et al. (2003).

#### Language input

Using an hour-by-hour questionnaire from 7 am to 11 pm, parents reported on the English and Spanish or Chinese child input at home on a typical day of the week and a typical weekend. In this part of the survey, parents were asked about the specific activities that their child regularly does, who they are usually with (adult or peers), and whether they are exposed to English, the HL, or both. The amount of exposure to English or the corresponding HL was calculated in percentages. This section of the parent questionnaire was adapted from the Bilingual Input–Output Survey (BIOS; Gibson et al., 2014).

#### DLLs’ expressive vocabulary

This study employed the Picture Vocabulary subtest from the Woodcock-Johnson IV Tests of Oral Language (Schrank et al., 2014). Validated parallel measures were used for English and Spanish. The Spanish version was translated into Chinese, as has been done in previous studies (e.g., Uchikoshi, 2013; Chernoff et al., 2021; Uchikoshi et al., 2022; Mak et al., 2023). Due to the lack of a normed Chinese version of the test, raw scores were used for all three languages. Children were asked to verbally provide a word for each picture shown. The median test reliability for English at age four is 0.94 (McGrew et al., 2014) and for Spanish at age four is 0.89 (Wendling et al., 2019). The alpha reliability in Chinese for our sample was 0.91.

### Data analysis

First, descriptive analyses of the study variables were conducted, and 2-sample *Z* tests of proportions were run to analyze the differences in reasons for migration between the two cultural groups. Second, independent ANOVAs and zero-order correlations were performed to investigate the relationships among reasons for migration, parental acculturation, child language input, and home literacy practices. Finally, following Streiner (2005) guidelines for model fit and model specification, two path analysis models were conducted for each cultural group. One model contained English home literacy practices and English language input as mediators of parents’ cultural orientations and DLLs’ English expressive vocabulary. The other model had Spanish/Chinese home literacy practices and Spanish/Chinese language input as mediators of parents’ cultural orientations and DLLs’ Spanish/Chinese expressive vocabulary. In testing the path models, all parameters were estimated freely. Following Streiner (2005) criteria for a good fit between the data and hypothesized models, the model χ^2^ statistic and fit indices (*χ*^2^ or chi-square ≥ 0.05, CFI ≥ 0.90, RMSEA ≤0.08, and SRMR ≤0.08) were used. The sample size to parameter ratio was 64:11 for the Mexican American group and 124:11 for the Chinese American group. All analyses were conducted using RStudio Version 1.4.1717 (RStudio Team, 2021) and the *tidyverse* (Wickham et al., 2019), *lavaan* (Rosseel, 2012), and *bcaboot* (Efron and Narasimhan, 2020) packets.

## Results

The descriptive statistics of the study variables for Mexican American and Chinese American families are shown in Table 2. No significant differences were found for any of the study variables across the two cultural groups.

**Table 2:** Descriptive statistics of the study variables.

	Mexican American (*n* = 66)				Chinese American (*n* = 124)				*M*	SD	Min	Max	*M*	SD	Min	Max
Parent cultural orientation								
American	2.2	0.79	1	4.22	2.22	0.71	1	4.31
Mexican/Chinese	3.53	0.51	2.44	4.56	3.27	0.61	1.89	4.89
Home literacy								
English	3.70	1.08	1	5	3.46	1.20	1	5
Spanish/Chinese	3.50	1.31	1	5	3.20	1.36	1	5
Language input %								
English	32.41	21.95	0	97.50	33.61	14.92	10	70.50
Spanish/Chinese	52.17	20.77	7.50	99	51.68	16.44	10.50	99
Child expressive vocab.								
English (raw)	11.17	5.78	0	22	12.22	5.79	0	28
Spanish/Chinese (raw)	10.75	7.22	0	22	10.98	6.63	0	23

### Chinese and Mexican parents’ reasons for migration

As displayed in Table 3, results revealed that most Mexican parents (48.48%) migrated for “multiple” reasons (i.e., both “family” and “betterment” reasons), while the majority of Chinese parents (71.77%) migrated to the U.S. solely for “family” reasons. Regarding the differences between the two cultural groups, 2-sample Z tests of proportions indicated that Chinese parents (71.77%, *M* = 0.58) migrated to the U.S. exclusively for “family” reasons significantly more than Mexican parents (13.64%, *M* = 0.21), *p* = 1.97e-06, whereas Mexican parents (16.67%, *M* = 0.25) migrated to the U.S. solely for “betterment” reasons significantly more than their Chinese counterparts (8.87%, *M* = 0.07), *p* = 0.001114. Additional reasons for migration reported by Mexican parents under the “other” category were: “to look for a better future,” “to send money to Mexico to help my family,” “because I felt threatened in Mexico,” “to help my sister,” and “because of neighborhood violence.” Under the “other” category, three Chinese parents reported migrating to the U.S. “to find better and more accessible education.”

**Table 3:** Reasons for migration among Mexican and Chinese parents.

	Mexican American (*n* = 66)		Chinese American (*n* = 124)	
Migration reason	Number	Percentage	Number	Percentage
Family	9	13.64	89	71.77
Betterment	11	16.67	11	8.87
Multiple	32	48.48	18	14.52
NA	14	21.21	6	4.84

The category “multiple” is a combination of “family” and “betterment” reasons for migration. The label “NA” applies to unspecified reasons or missing answers.

### Reasons for migration and acculturation

The categorical variable “reasons for migration” was only significantly associated with American cultural orientation among Mexican American families [*t*(2) = 5.21, *p* = 0.009], and it was not significantly associated with either home literacy practices or language input in any language for both cultural groups. Tukey post-hoc tests showed that Mexican families who migrated for “betterment” reasons (e.g., looking for a better job or education opportunities for them and/or their children) reported significantly higher levels of American cultural orientation than families who migrated solely for “family” reasons (e.g., meeting with family members living in the U.S., getting married), *p* = 0.007. Families who migrated for “multiple” reasons also reported significantly higher levels of American cultural orientation than families who migrated exclusively for “family” reasons, *p* = 0.032. No differences in American or heritage cultural orientations were found between families who migrated for “multiple” reasons and “betterment” reasons, *p* > 0.05.

### Pathways predicting children’s expressive vocabulary

Since no significant, direct relationships between parents’ American and Mexican/Chinese cultural orientations and children’s English and HL expressive vocabulary were found, four models were used to estimate the paths from parents’ cultural orientations to children’s English and Spanish/Chinese vocabulary separately via home literacy practices and language input in both languages for both cultural groups. The path analysis models predicting DLLs’ expressive vocabulary skills in English did not fit the data for either the Mexican American or the Chinese American families. Therefore, these models are not reported in this paper. Conversely, the path analysis models predicting DLLs’ expressive vocabulary in their HL (Spanish/Chinese) fit the data for both cultural groups.

**Figure 1:**
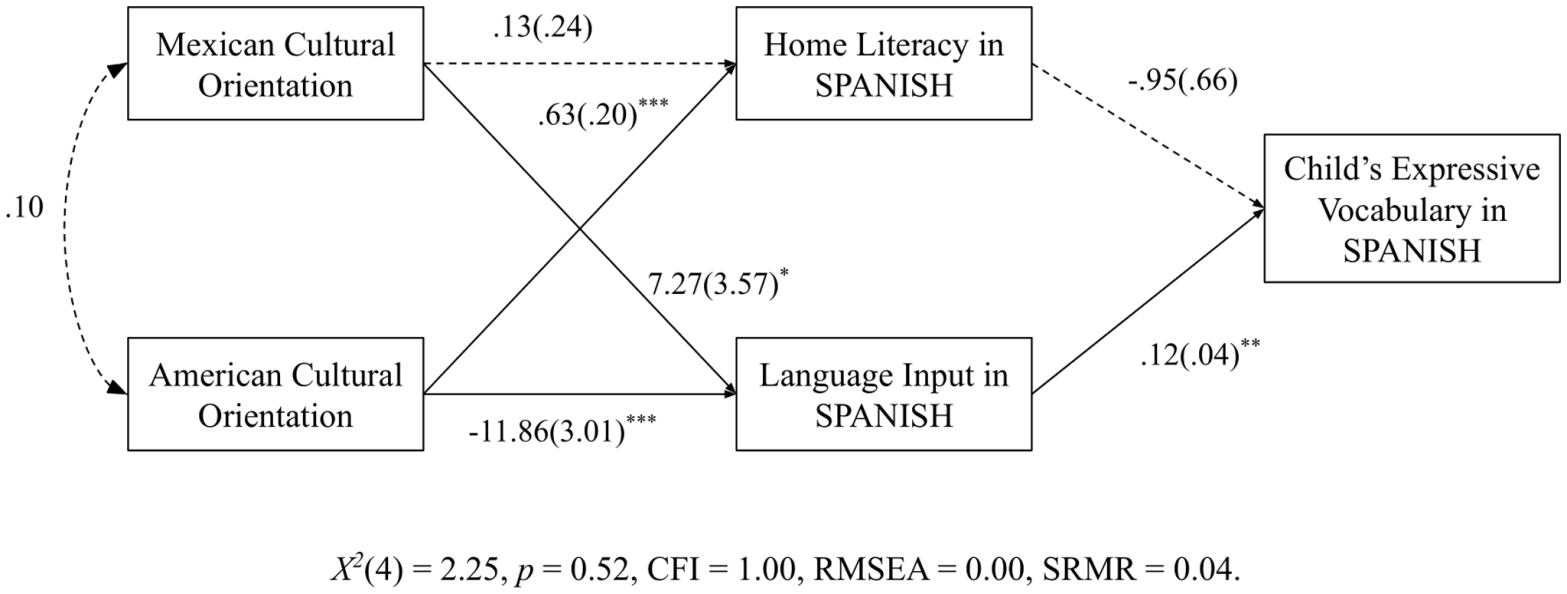
Pathways to Mexican-American DLLs’ expressive vocabulary (*N* = 66).

In the model where home literacy practices and language input in Spanish were the outcome variables, results showed that language input was positively predicted by Mexican cultural orientation (*b* = 7.27, *p* = 0.05) and negatively predicted by American cultural orientation (*b* = −18.86, *p* = 0.001). In turn, language input in Spanish positively predicted DLLs’ expressive vocabulary in Spanish (*b* = 0.12, *p* = 0.01). However, home literacy in Spanish did not significantly predict children’s vocabulary in Spanish. For Mexican American families, language input in Spanish was found to mediate the relationship between parents’ American and Mexican cultural orientations and DLLs’ Spanish expressive vocabulary, *X*^2^(4) = 2.25, *p* = 0.52, CFI = 1.00, RMSEA = 0.00, SRMR = 0.04. The model ran without errors and evidenced good fit to the data. *Post hoc* mediation analyses were conducted to test whether language input in Spanish significantly mediated the relationship between parents’ cultural orientations and DLLs’ expressive vocabulary in Spanish. Indirect effects were tested using the bias-corrected bootstrap confidence interval (BCBCI) approach (MacKinnon et al., 2004; Efron and Narasimhan, 2020), and two significant indirect effects were found (see Figure 1): (a) Mexican cultural orientation → Language input in Spanish → Child’s expressive vocabulary in Spanish, 95 CI [−0.5781, 1.7251]; and (b) American cultural orientation → Language input in Spanish → Child’s expressive vocabulary in Spanish, 95 CI [−2.589, −0.106].

**Figure 2:**
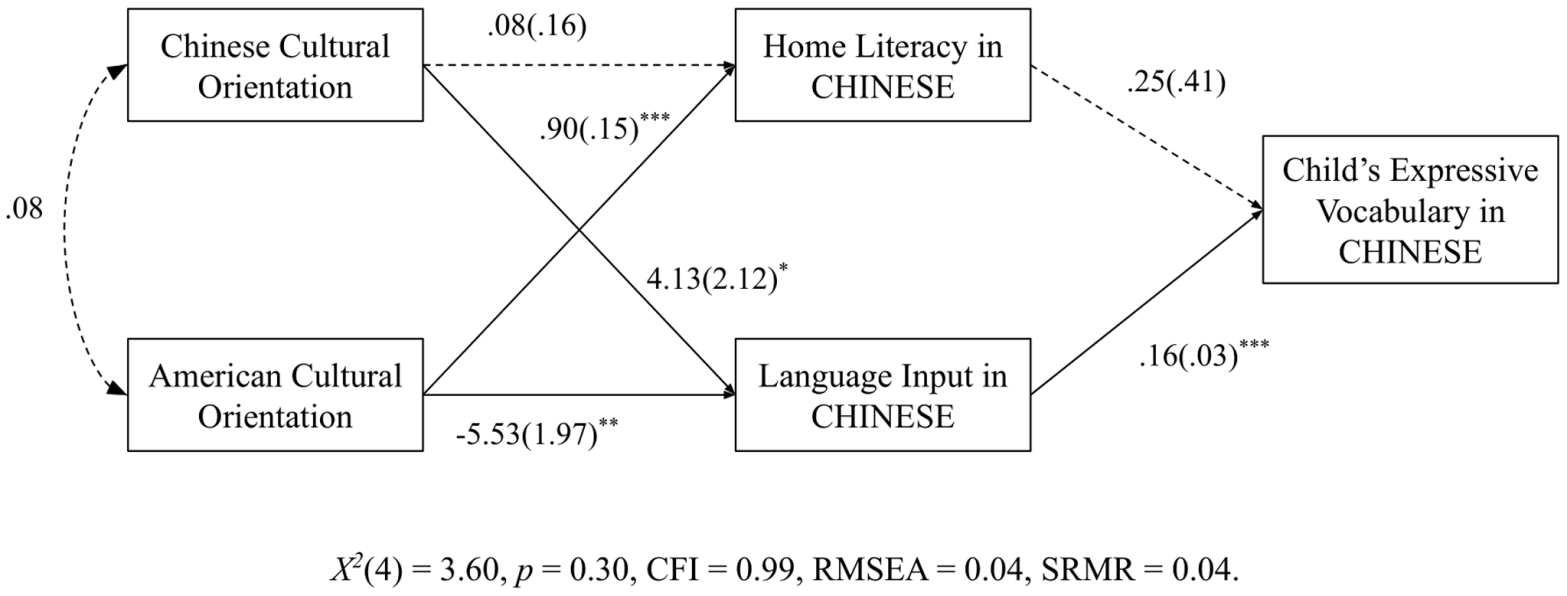
Pathways to Chinese-American DLLs’ expressive vocabulary (*N* = 124).

In the model where home literacy practices and language input in Chinese were the intervening variables, results showed that language input was positively predicted by Chinese cultural orientation (*b* = 4.13, *p* = 0.05) and negatively predicted by American cultural orientation (*b* = −5.53, *p* = 0.01). In this model, language input in Chinese positively predicted DLLs’ Chinese expressive vocabulary (*b* = 0.16, *p* = 0.001). However, home literacy in Chinese did not significantly predict DLLs’ Chinese vocabulary. Hence, for Chinese American families, language input in Chinese was also found to mediate the relationship between parents’ American and Chinese cultural orientations and DLLs’ Chinese expressive vocabulary, *X^2^*(4) = 6.54, *p* = 0.16, CFI = 0.94, RMSEA = 0.07, SRMR = 0.05. The model ran without errors and evidenced good fit to the data. *Post hoc* mediation analyses were conducted to test whether language input in Chinese significantly mediated the relationship between parents’ cultural orientations and DLLs’ expressive vocabulary in Chinese. Indirect effects were tested using the BCBCI approach. As in the previous model, two significant indirect effects were found (see Figure 2): (a) Chinese cultural orientation → Language input in Chinese → Child’s expressive vocabulary in Chinese, 95 CI [−0.4015, 1.2792]; and (b) American cultural orientation → Language input in Chinese → Child’s expressive vocabulary in Chinese, 95 CI [−1.4084, −0.0475].

## Discussion

The purpose of the present study was to characterize the reasons for migration and examine the relations among parental acculturation, home literacy practices, language input, and DLLs’ expressive vocabulary in a group of 190 Mexican American and Chinese American parent–child dyads living in Northern California. Regarding the reasons for migration across the two cultural groups, we found that the majority of Mexican American families migrated to the U.S. for “family” or “betterment” reasons, whereas most of the Chinese American families in our study migrated to the U.S. solely for “family” reasons. Regarding the associations between reasons for migration and parental acculturation, home literacy practices, and children’s language input, we found that the variable “reasons for migration” was only significantly associated with parents’ American cultural orientation among Mexican American families. Specifically, the results revealed that Mexican families who migrated for “betterment” or “multiple” reasons are more likely to be more acculturated to the receiving culture (i.e., the U.S. American Culture) than families who migrated for “family” reasons, who may tend to retain more values and behaviors from their culture of origin (i.e., the Mexican culture). The findings for the third research question on the role of parental acculturation, home literacy practices, and language input on DLLs’ expressive vocabulary showed that for both cultural groups, child language input mediated the relationship between parents’ cultural orientations and DLLs’ expressive vocabulary in their HL. In particular, parents’ Mexican/Chinese cultural orientations positively predicted Spanish/Chinese input, which in turn predicted children’s Spanish/Chinese vocabulary. In these models, home literacy in the HL was positively predicted by parents’ American cultural orientation but did not significantly predict DLLs’ HL vocabulary skills in preschool. A potential explanation could be that the more schools promote home literacy practices, and the more parents relate to the American school system, the more they tend to engage in home literacy practices in their HL. It is possible that literacy practices did not significantly predict DLLs’ vocabulary in our models because other variables are needed to explain the wide range of variation in bilingual preschoolers’ oral language. A possible explanation as to why the models predicting children’s English vocabulary with English home literacy and English language input as intervening variables did not fit the data could be model misspecification, i.e., other variables that play a major role in DLLs’ English vocabulary were not specified or included in the models (Streiner, 2005).

Based on the information available, our study is the first one to investigate the reasons for migration among low-income immigrant parents of young DLLs living in the U.S. This study is also the first one to simultaneously study two low-income immigrant groups from different languages and cultural backgrounds. Research has shown that Asian Pacific- and Latino-origin individuals tend to place a higher value on familism when compared to the more individualistic U.S. culture and cultural values (e.g., Tseng, 2004; Stein et al., 2020). This can help us explain why both cultural groups participating in our study reported “family” as their main reason for migration. However, since previous studies have been conducted with early and late adolescents, comparisons between the results warrant caution. Additionally, our results partially align with previous research on Asian immigrants living in the U.S. (Lobo and Salvo, 1998; Chen et al., 2009). The differences lay in that, besides family reasons, and educational and employment opportunities, these studies reported that a smaller number of Chinese families migrated to the U.S. for political refuge on the 1990s. In line with a recent study, we observed that joining family members in the U.S. was Chinese families’ main reason for migration (Wang et al., 2023). Yet, Wang and colleagues investigated migration reasons among low-income (50%) and middle- to higher-income (50%) Chinese American families, whereas, in this study, we characterized the reasons for migration among low-income (100%) Chinese American and Mexican American families. This may explain why almost one quarter of the population sampled by Wang et al. migrated for employment-based reasons, whereas most immigrants in our study migrated for family reasons, and little variability was observed within and across the two cultural groups. Nevertheless, it is important to remember that motivations and reasons for migration are complex, as they may vary within and across cultural groups and be linked to several socioeconomic and political factors (e.g., Martin et al., 2006; Czaika and Reinprecht, 2022).

There are a few possible explanations as to why Mexican families who migrated for “betterment” or “multiple” reasons are more likely to be more acculturated to the American culture than families who migrated solely for “family” reasons. First, families who migrated to get a better job or education opportunities may be more immersed in job and/or school domains where English is the most frequently used language and where individuals are more exposed to norms and expectations in the U.S. This may positively influence their American cultural orientation. Second, families who migrated mainly or solely for family reasons may have more social connections from their heritage culture (e.g., family members and friends), which may positively influence their heritage culture cultural orientation. Concurrently, these families may also place a higher value on familism, as shown above, leading them to stay more connected to their cultural roots and values. Third, it is possible that these families achieved a higher level of English proficiency in their home country, which might have paved the way for more job opportunities and social interactions with non-Mexican co-workers, easing the adaptation to the American culture. Another possible explanation is that since, at the time of data collection, Mexican American parents had lived in the U.S. for an average of six years more than Chinese American parents, the former might have had more time to adapt to the receiving culture, leading to higher levels of American cultural orientation.

Aligned with Tsai et al.’s (2012) results, our findings revealed that Chinese cultural orientation predicted parents’ Chinese language use (child language input), which in turn predicted DLLs’ Chinese expressive vocabulary. Cote and Bornstein (2014) also concluded that maternal acculturation predicted the amount of input in the HL and receiving culture’s dominant language, which then predicted children’s vocabulary size in both languages. However, this study was conducted with Latina immigrants (primarily from Argentina, Colombia, and Peru), Japanese immigrants, and Korean immigrants living in the U.S. To the best of our knowledge, our study is the first one to find and report similar results for a group of Mexican American parents of young children living in the U.S., for whom Mexican cultural orientation predicted parents’ Spanish language use, which in turn predicted DLLs’ Spanish expressive vocabulary in preschool. Hence, this study highlights the importance of language input in bilingual children’s early vocabulary development, especially in their HL (Hammer et al., 2014). Even though children’s HL reading and writing skills were not assessed, it is important to acknowledge the differences between Spanish as an alphabetic language and Chinese as a non-alphabetic language, especially when interpreting why home literacy practices in the HL were not significant in the pathways explaining DLLs’ expressive vocabulary. Since the frequency with which parents help their children with letters, numbers, and characters was one of the items measuring home literacy practices, we must consider that phonological decoding may look different for English-Spanish DLLs versus English-Chinese DLLs (Conrad, 2016).

The current study has implications for immigrant families, teachers, and DLL school administrators. The results can help inform stakeholders about the reasons for migration of the two largest cultural and linguistic groups in California, and the relationship among parents’ cultural orientation, language input, and DLLs’ HL vocabulary skills. Even though reasons for migration and acculturation strategies may vary within and across groups, some cultural groups may share certain characteristics (Berry, 2019). In early education contexts, it is important to remember that parental acculturation and reasons for migration can be linked to the child’s development, identity, social relationships, and overall school success (e.g., Bornstein and Cote, 2006). Thus, specific and culturally relevant aspects of parental acculturation can inform instructional approaches and curriculum design that consider linguistically and culturally diverse learners. The significant relationships between parents’ cultural orientations and child language input emphasize the importance of understanding the socialization practices, media usage, and language practices of immigrant parents and their young children (e.g., Tsai et al., 2012; Troesch et al., 2021). Finally, our results stress the importance of fostering HL use at home and in schools and its crucial role in HL maintenance. A deeper understanding of diverse parental acculturation practices can help educators better support children from diverse backgrounds as well as promote cultural awareness and sensitivity in the classroom.

### Limitations and future directions

It is essential to acknowledge the following limitations. First, the variables “reasons for migration” and “parents’ cultural orientation” should be interpreted with caution given that they were measured using Likert scale surveys and multiple-choice questionnaires, which may have influenced or limited the participants’ responses. The “reasons for migration” category should also be carefully conceptualized given that a reason such as “providing children with better education and opportunities” could well fall under both “betterment” and “family” reasons. For example, in some collectivistic cultures (e.g., Chinese) children’s academic success is an integral part of a family’s progress and prosperity. Migration drivers and acculturation processes are complex and difficult to accurately represent with one or a few statements. It is usually “a number of factors that are mutually mediating and conjointly shaping migration decisions and broader migration dynamics and patterns” (Czaika and Reinprecht, 2022, p. 50). Thus, future research should incorporate qualitative data such as interviews or *testimonios* to better understand immigrant parents’ migration and acculturation experiences during a sense-making process. Second, the Mexican American sample in this study was relatively smaller than the Chinese American sample. Future investigations should ideally examine larger groups of parent–child dyads to increase the statistical power of the path analysis models. Third, the results obtained cannot establish any causal relationships due to the non-experimental design of the study. Upcoming studies should investigate the causal relationships between parental acculturation, language input, and DLLs’ language outcomes. Despite the limitations, our findings provide new insights, showing that the various ways Mexican American and Chinese American families acculturate to the U.S. and retain values from their cultures of origin can relate to their language use and the HL development of DLLs.

## Conclusion

With almost one-quarter of the total population of parents being immigrant parents of children ages 0 to 10 in the U.S. (Hofstetter and McHugh, 2021), it is of utmost importance for teachers to engage with immigrant families and for families to have support while navigating a new culture, language, and school system. Because immigrant parents play a major in transmitting their heritage language and values to their children (e.g., Bornstein, 2009; Baker, 2016), a better understanding of their cultural orientations and language use can help educators better assist their culturally and linguistically diverse students, all of whom should be encouraged to maintain their HL and see their bilingualism as an invaluable asset.

## Data availability statement

The raw data supporting the conclusions of this article will be made available by the authors, without undue reservation.

## Ethics statement

The study was reviewed and approved by Institutional Review Boards (IRBs) at the University of California, Berkeley and the University of California, Davis. Written informed consent to participate in this study was provided by the adult participants (i.e., child’s legal guardian or next of kin).

## Author contributions

MB, QZ, and YU contributed to the conception and design of the study. MB organized the database, performed the statistical analyses, and wrote the first draft of the manuscript. All authors contributed to manuscript revision, read, and approved the submitted version.

## Funding

This study and the project *Language Emotion and Development* (LEAD) at large were funded by the NIH/NICHD (R01HD091154): “Bilingual and Socio-Emotional Development in Dual Language Learners”.

## Conflict of interest

The authors declare that the research was conducted in the absence of any commercial or financial relationships that could be construed as a potential conflict of interest.

## Publisher’s note

All claims expressed in this article are solely those of the authors and do not necessarily represent those of their affiliated organizations, or those of the publisher, the editors and the reviewers. Any product that may be evaluated in this article, or claim that may be made by its manufacturer, is not guaranteed or endorsed by the publisher.

## Footnotes

^1^This study will use the terms “acculturation” and “cultural orientation” interchangeably, with the assumption that acculturation is a two-way process that involves cultural orientation toward the culture of origin and cultural orientation toward the receiving culture.
